# Identification of T cell stress response state (T_STR_) and key genes related to T cells in discoid lupus erythematosus

**DOI:** 10.1038/s41598-025-23088-7

**Published:** 2025-11-10

**Authors:** Xingfeng Zhuo, Xue Xing, Kai Xing, Li Zhang, Yong Wang, Yao Yao, Xia Li, Qian Li, Nan Chen, Chunxia Chen, Xiaoling Yu, Juanjuan Peng, Lei Zhang

**Affiliations:** 1 Jinling Hospital, Affiliated Hospital of Medical School, Nanjing University, Nanjing, 210000 China; 2 School of Early-Childhood Education, NanJing XiaoZhuang University, Nanjing, 210000 China

**Keywords:** Cell biology, Biomarkers, Molecular medicine

## Abstract

The malignant transformation risk and high misdiagnosis rate of discoid lupus erythematosus (DLE) render it an urgent necessity to deeply explore its pathogenesis and biomarkers. This research, through the combined analysis of transcriptome data and single-cell sequencing data, commencing from the highly infiltrated T cells in DLE and the significant inflammation and cytokine correlation manifested by T cells, undertakes re-clustering analysis of T cells and identifies the stress response state T cells (T_STR_) that are abundantly infiltrated in the dermis of DLE. Furthermore, by screening for the common genes among the differentially expressed genes in the transcriptome, the differentially expressed genes of T cells in single-cell sequencing, and the key module genes in WGCNA, CXCL13, GNLY, IFI6, IFI27, IFI44, IFI44L, MX1, and TIGIT are ultimately extracted as the key disease genes of DLE, and these genes are closely associated with the mechanism regulating T cell function. Specifically, through supplementary validation of gene expression using the psoriasis dataset, the distinctively high expression characteristics of CXCL13 and GNLY in DLE were revealed, thereby corroborating their central status as characteristic markers of DLE.

**Supplementary Information:**

The online version contains supplementary material available at 10.1038/s41598-025-23088-7.

## Introduction

Discoid lupus erythematosus (DLE) represents the most prevalent type among chronic cutaneous lupus erythematosus. Simultaneously, it harbors the risk of malignant transformation. Research indicates that long-term DLE may undergo malignant conversion into cutaneous squamous cell carcinoma (cSCC), and this type of cancer is highly invasive, prone to early metastasis, and has a relatively high mortality rate^1,2^. On the other hand, in clinical diagnoses, DLE is prone to being misdiagnosed as psoriasis or seborrheic dermatitis, resulting in the inability to receive timely and correct treatment^3^. Hence, identifying more biomarkers capable of facilitating early detection is indispensable for achieving precise and scientifically accurate diagnoses and developing more intervention strategies.

At present, numerous studies have disclosed that immune system dysregulation serves as the crucial mechanism underlying the onset and progression of DLE. The abnormal activation of immune cells prompts the excessive secretion of inflammatory factors (such as IL-17) and the generation of autoantibodies (such as antinuclear antibodies and anti-Ro/SSA antibodies), thereby inducing inflammation and damage to the self-skin tissue^4^. Furthermore, long-term skin inflammation might cause abnormal repair of DNA damage, thereby augmenting the risk of abnormal cell proliferation^5^. Additionally, these epigenetic alterations may facilitate disease progression. For example, it has been verified that two CpG sites within the IFI44L promoter are significantly hypomethylated in patients with systemic lupus erythematosus (SLE)^6^. Concurrently, the chronic inflammatory environment is more favorable for immune tolerance, further magnifying the autoimmune response^7^.

T-cell dysfunction and T-cell cytotoxic responses play a crucial role in the pathogenesis of DLE. Research indicates that there exists a more pronounced pro-inflammatory and regulatory imbalance in DLE, and this reaction is positively correlated with CD25(+)/Foxp3(+) Treg cells^8^. Furthermore, upon detection, a greater number of CD4(+) T cells, Granzyme B(+) cells, and PD-1(+) cells are present in the dermis of DLE patients, suggesting that T-cell cytotoxic responses play a key role in the onset of DLE^9^. Single-cell transcriptomic analyses also demonstrate that T cells in the skin lesions of DLE patients lack large-scale activation, but the expression of cytotoxicity-related genes (such as perforin and granzyme) is relatively low, characterized by low cytotoxicity and a higher proportion of memory T cells^10^. Additionally, the infiltration of immune cells in the dermis of DLE patients (particularly T and B cells) is significantly elevated, and it has been discovered that in the Tph (peripheral helper T cell) and Tfh (Follicular helper T cell) cell clusters, co-stimulatory genes such as ICOS and TIGIT, as well as HLA-DRA and the transcription factor MAF, are upregulated, and these molecules may be associated with the mechanism of T/B cell interaction and the exacerbation of DLE skin inflammation^11^ (The samples used in the above-mentioned studies were all skin biopsy specimens from healthy individuals and patients.).

Currently, the rapid advancement of bioinformatics sequencing techniques has significantly propelled the research progress of DLE. Simultaneously, at the cellular or molecular levels, it is conducive to precisely differentiating the subtypes of lupus erythematosus. Some researchers have discriminated DLE from subacute cutaneous lupus erythematosus (SCLE) based on the variance in B-cell infiltration^12,13^; in the disparity research between psoriasis and DLE, the bias in the phenotypes of Th1 and Th17 was employed as the cellular characteristic of DLE^14^. Furthermore, there are studies suggesting that the methylation of IFI44L and the concentration of IFN-α1 in serum might be utilized as biomarkers for differentiating DLE from SLE^15^.

In this study of ours, transcriptomics and single-cell sequencing were combined for multi-omics integration, with the aim of exploring the characteristics of immune cell infiltration in the pathogenesis of DLE through analytical approaches such as differential analysis, immune infiltration analysis, enrichment analysis, Weighted Gene Co-expression Network Analysis (WGCNA), and correlation analysis. With the major infiltrating cells (T cells) as the in-depth focus, re-clustering analysis was carried out to reveal the main cellular characteristics during the pathogenesis of DLE. Furthermore, through the overlapping screening of differentially expressed genes in multiple analyses, biomarkers related to the major infiltrating cells (T cells) were ultimately selected.

## Methods

### Data collection

The data employed in our research were all derived from the public datasets of the NCBI Gene Expression Omnibus (GEO) (https://www.ncbi.nlm.nih.gov/geo/). Among them, the single-cell sequencing dataset GSE179633^11^ collected skin tissues of 5 healthy individuals (HC) and 8 patients with DLE, from which 4 HC epidermal single-cell suspensions, 5 DLE epidermal single-cell suspensions, 4 HC dermal single-cell suspensions, and 5 DLE dermal single-cell suspensions were prepared. The transcriptome data utilized three datasets, GSE81071, GSE184989 and GSE52471. GSE81071 collected skin tissues of 7 healthy individuals and skin tissues of 26 patients with DLE^16^, GSE184989 collected skin tissues of 4 healthy individuals and skin tissues of 26 patients with DLE, while GSE52471 collected skin tissues of 3 healthy individuals, skin tissues of 11 patients with DLE and skin tissues of 5 patients with psoriasis.

### Transcriptome data analysis

This study was based on the datasets GSE81071 and GSE184989. The R toolkit Limma was employed for screening differentially expressed genes, with the significance threshold set at Pvalue < 0.05 and |log2(FoldChange)| > 0.5. Gene Ontology Enrichment Analysis (GO) was carried out to perform functional annotation of the differentially expressed genes, encompassing three major categories: biological processes (BP), molecular functions (MF), and cellular components (CC). Simultaneously, the Kyoto Encyclopedia of Genes and Genomes (KEGG) database was utilized to undertake enrichment studies of metabolic pathways. During the analysis process, the R toolkit clusterProfiler was used to accomplish functional annotation and pathway enrichment calculations, and the R toolkit ggplot2 was employed to visualize the results. Additionally, the Cibersort algorithm^17^ was applied to quantify the differences in the infiltration levels of 22 types of immune cells between the healthy group (Normal) and the DLE patient group (DLE).

### Weighted gene Co-expression network analysis (WGCNA)

WGCNA identifies potential biomarker genes or therapeutic targets based on the internal connectivity of gene sets and the association between gene sets and phenotypes^18,19^. To determine the gene modules most relevant to the pathogenesis of DLE, the datasets GSE81071 and GSE184989 were initially integrated, and the top 75% of highly variable genes (MAD ≥ 0.01) were screened for subsequent analysis. The R package WGCNA was utilized to determine the most appropriate soft threshold β for network construction by computing the scale-free topology fitting index for each power, ensuring that the scale-free topology fitting index approaches 0.9, in order to construct a co-expression network that satisfies self-similarity. A power function transformation was employed to generate a weighted adjacency matrix, and the topological overlap matrix (TOM) was further calculated to mitigate noise interference. Hierarchical clustering was adopted to identify gene modules, and modules with a similarity exceeding 50% were merged. The expression profiles of each module were characterized by module eigengenes, and the correlation coefficient and statistical significance with the DLE phenotype were calculated. Modules with an integrated connectivity (eigengene) exceeding 0.75 were integrated to identify the modules most relevant to the characteristics of DLE, and a heatmap of inter-module correlations was plotted to showcase the network structure.

### Single-cell RNA-seq data processing and analysis

Obtain the matrix information of the dataset GSE179633 and conduct unsupervised clustering of cells using the R package Seurat. Filter out cells expressing more than 300 and less than 8000 genes, with the proportion of red blood cells being less than 5% and that of mitochondria being less than 10%. Utilize the “FindIntegrationAnchors” and “IntegrateData” functions for sample integration, setting the dims parameter to 20. Subsequently, perform principal component analysis (PCA), construct a SNN network using the top 10 PCs, and identify cell clusters with a resolution of 0.5 using the Louvain algorithm. Finally, apply UMAP to visualize the clustering results in a two-dimensional space. In this study, the following cell marker genes were selected as markers for each cell cluster annotation, and UMAP feature maps [Supplementary Fig. 1] were used to display the expression of marker genes^11,20^: B (CD79A, MS4A1), Endothelials (MMRN1, SELE), Fibroblasts (LUM, PRRX1), Keratinocytes (DSG1, LGALS7B), Macros/DCs (C1QC, LYZ), Masts (CPA3, TPSB2), Melanoocytes (MLANA, TYRP1), NKs (GNLY, XCL1), Schwanns (MPZ, NRXN1), T (CD3D, CD8A). The expression of some marker genes in the initial unannotated clusters of the T cell cluster after re-clustering was displayed using a bubble chart [Supplementary Fig. 2]: HSP_TSTR (HSPA6, DNAJB1), Treg (FOXP3, FOXO1, IL2RA, CTLA4, TNFRSF4), CD8Trm (XCL1, XCL2, GZMK), Tn (CCR7, TCF7, SELL), MT1E_Tm (MT1E, MT1X, MT2A), CD8Tem (GZMH, IFNG, CCL3, GZMA, CCL4, NKG7), Th17 (ANKRD28, CCR6, CAPG, IL22).

### Pseudotime analysis

Based on the single-cell sequencing dataset GSE179633, we employed the Monocle2 package in R language to carry out cell trajectory analysis^21^. Initially, the raw data was standardized, denoised, and clustered using the Seurat tool to generate a cell data object (CDS). Subsequently, the DDRTree algorithm was adopted to reduce the dimensionality of the cells, mapping the complex high-dimensional data into a two-dimensional space to facilitate subsequent analysis. Then, key genes (such as differentially expressed genes or highly variable genes) were screened to infer the differentiation status of the cells, and the “orderCells” function was utilized to sort the cells and simulate their developmental timeline. Eventually, the “plot_cell_trajectory” function was applied to generate visual results, presenting the cell differentiation trajectory and its evolutionary path.

### Venn diagram

The gene lists that are required to generate Venn diagrams are taken as the input data. The intersections of specific sets are extracted through logical operations. The R package VennDiagram is employed to draw the diagrams^22^. The venn.diagram() function is utilized to specify the grouping columns for statistics, perform automatic statistics and draw the Venn diagrams, and the get.venn.partitions() function is used to obtain the specific names of the intersection elements among each group.

### Statistical analysis

For differential expression analysis, we adopt Student’s T-test to compare whether there exists a significant difference in the means of two sample groups, which is applicable when the sample size is large and the data conform to a normal distribution. In cases where differences among multiple groups need to be compared, such as analyzing the gene expression between the DLE onset group and the healthy group, we utilize One-Way ANOVA to examine statistically significant differences among the groups. For the differential detection of immune infiltrating cells, we employ the Wilcoxon rank sum test, which does not require the data to follow a normal distribution and is suitable for situations with a small sample size or when the data distribution does not satisfy normality. Furthermore, a P-value (or adjusted P-value) less than 0.05 is defined as statistically significant. All statistical analyses were accomplished using R software and its related toolkits.

## Result

### The imbalance of immune homeostasis and the activation of inflammatory cells in DLE

We separately performed transcriptome analyses on the two collected microarray datasets, GSE81071 and GSE184989, respectively, as mutual validations to guarantee the accuracy of our bioinformatics analysis. The sample groupings in both microarray datasets were the healthy control skin biopsy group (HC) and the discoid lupus erythematosus patient skin biopsy group (DLE).

**Fig. 1:**
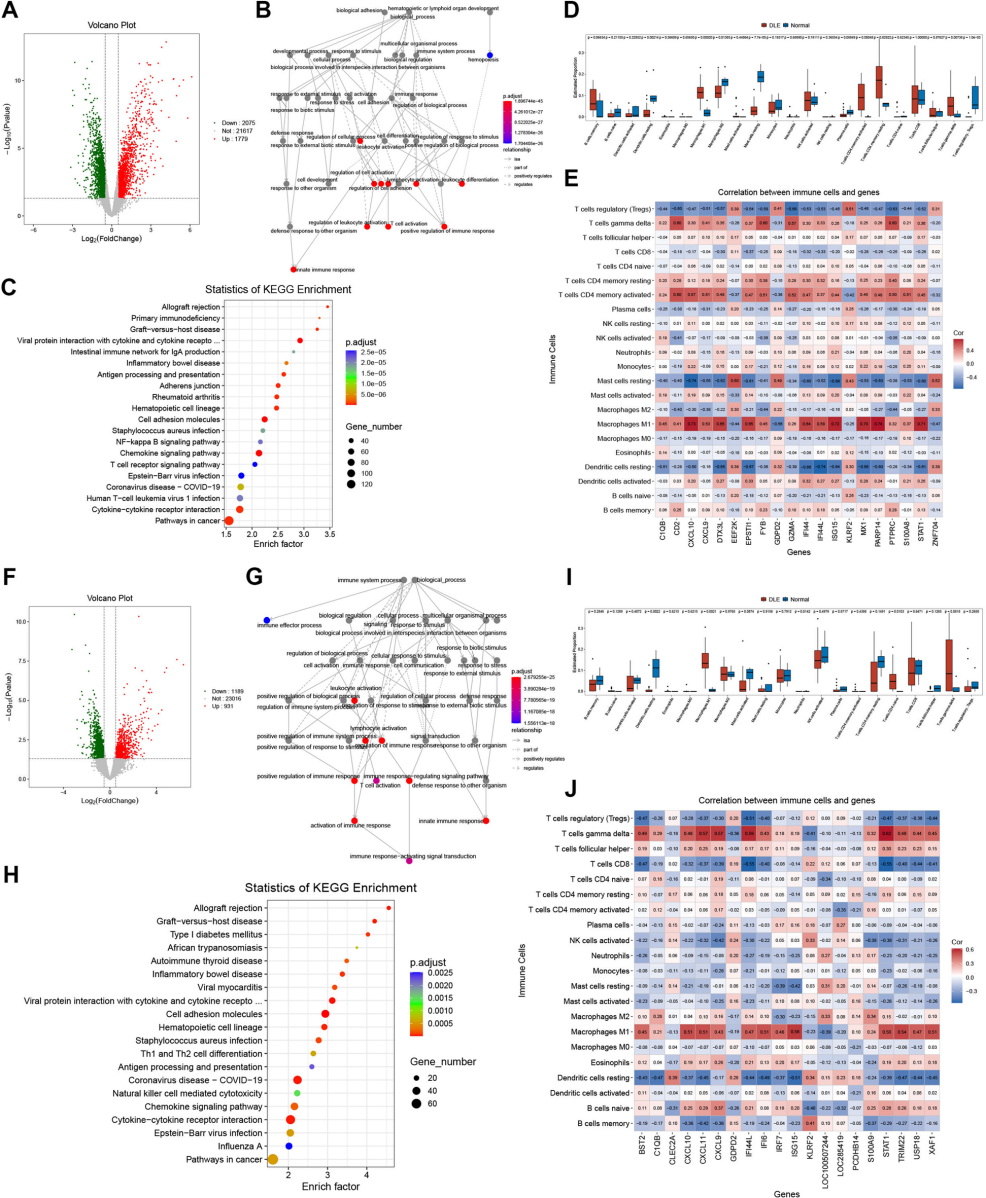
**The analytical outcomes of transcriptome data based on GSE81071 (A-E) and GSE184989 (F-J). A** The volcano plot of differentially expressed genes between the HC and DLE groups in GSE81071 (with Pvalue < 0.05 and |log2FC| > 0.5), where each green dot indicates a down-regulated gene and each red dot indicates an up-Regulated gene. **B** Based on the differentially expressed genes in A, a GO enrichment network graph was constructed. The color of each point indicates the enrichment significance of the corresponding GO entry. The arrow shapes between points, as depicted in the legend, represent the relationship between two GO entries. **C** Based on the differentially expressed genes in A, a KEGG enrichment bubble plot was generated. The size of the dots represents the number of enriched genes in the pathway, and the color of the dots indicates the enrichment significance of the pathway. **D** The box plot for immune infiltration analysis based on GSE81071, where the x-axis represents the names of the infiltrating cells and the y-axis represents the infiltration proportion. The infiltration differences of each immune cell between the HC and DLE groups have undergone statistical analysis, and the P values are indicated. **E** The correlation heatmap between 22 types of immune cells and infiltration-related genes based on GSE81071. **F** The volcano plot of differentially expressed genes between the HC and DLE groups in GSE184989. **G** Based on the differentially expressed genes in F, a GO enrichment network graph was generated. **H** Based on the differentially expressed genes in F, a KEGG enrichment bubble plot was generated. **I** The box plot for immune infiltration analysis based on GSE184989. **J** The correlation heatmap between 22 immune cell types and infiltration-related genes based on GSE184989.

Based on the microarray dataset GSE81071, we conducted a differential analysis between the HC and DLE groups (with the threshold set as Pvalue < 0.05 and |log2FC| > 0.5), and collected 2,075 down-regulated genes and 1,779 up-regulated genes [Fig. 1A] [Supplementary Table S1]. The biological processes related to these differentially expressed genes were concentrated in processes such as “T cell activation” and “innate immune response” [Fig. 1B], suggesting that the occurrence and development of DLE are always closely associated with the dynamic response of the immune system, and the state of T cells might play a crucial role in this process. Subsequently, we performed KEGG pathway enrichment analysis on these differentially expressed genes. Excluding some irrelevant disease pathways, we discovered that the most significantly enriched differential pathways were related to the interaction among cytokines (such as “Cytokine-cytokine receptor interaction”) and T cells (such as “T cell receptor signaling pathway”) [Fig. 1C]. Since these results indicated the significance of immune-related aspects, we further carried out immune infiltration analysis on the dataset GSE81071. The box plot presented the analysis results of 22 types of immune cells [Fig. 1D], showing a significantly increased infiltration of T cell populations in the DLE group, with T cells CD4(+) memory activated being the most prominent (*P* = 0.00048), while the infiltration of T cells regulatory significantly decreased in the DLE group, suggesting a disorder of T cell homeostasis during the development of DLE; moreover, the significantly increased infiltration of Macrophages M1 in the DLE group (*P* = 0.00020) also indicated the inflammatory invasion during the progression of DLE. Simultaneously, a correlation analysis was conducted on some genes related to immune cell infiltration [Fig. 1E]. Similar to the results of immune cell infiltration proportions, Macrophages M1 showed the most significant correlation with these genes, followed by T cells CD4(+) memory activated and T cells gamma delta.

The differential analysis between the HC and DLE groups based on the microarray dataset GSE184989 ultimately screened and retained 1,189 down-regulated genes and 931 up-Regulated genes [Fig. 1F] [Supplementary Table S2]. Similar to the analysis results of the dataset GSE81071, the differentially enriched GO terms encompassed “T cell activation”, “innate immune response”. [Fig. 1G]; the differentially enriched pathways were also concentrated in pathways such as “Cytokine-cytokine receptor interaction”, “Th1 and Th2 cell differentiation”. [Fig. 1H]. The results of the immune infiltration analysis in this dataset were not markedly different from those of the dataset GSE81071, but exhibited a similar trend, particularly for Macrophages M1 (*P* = 0.0021) and T cells CD4(+) naive (*P* = 0.00103) [Fig. 1I]. Furthermore, the correlation analysis with infiltration genes presented the same outcomes as the dataset GSE81071 [Fig. 1J], with the most significantly correlated cell clusters concentrated in Macrophages M1 and T cells gamma delta. In conclusion, both the differential enrichment analysis and the immune infiltration analysis based on the microarray datasets GSE81071 and GSE184989 indicate the imbalance of immune homeostasis and the activation of inflammatory cells during the development process of DLE.

### T-cell homeostasis serves as the key in the immune regulation of DLE

**Fig. 2:**
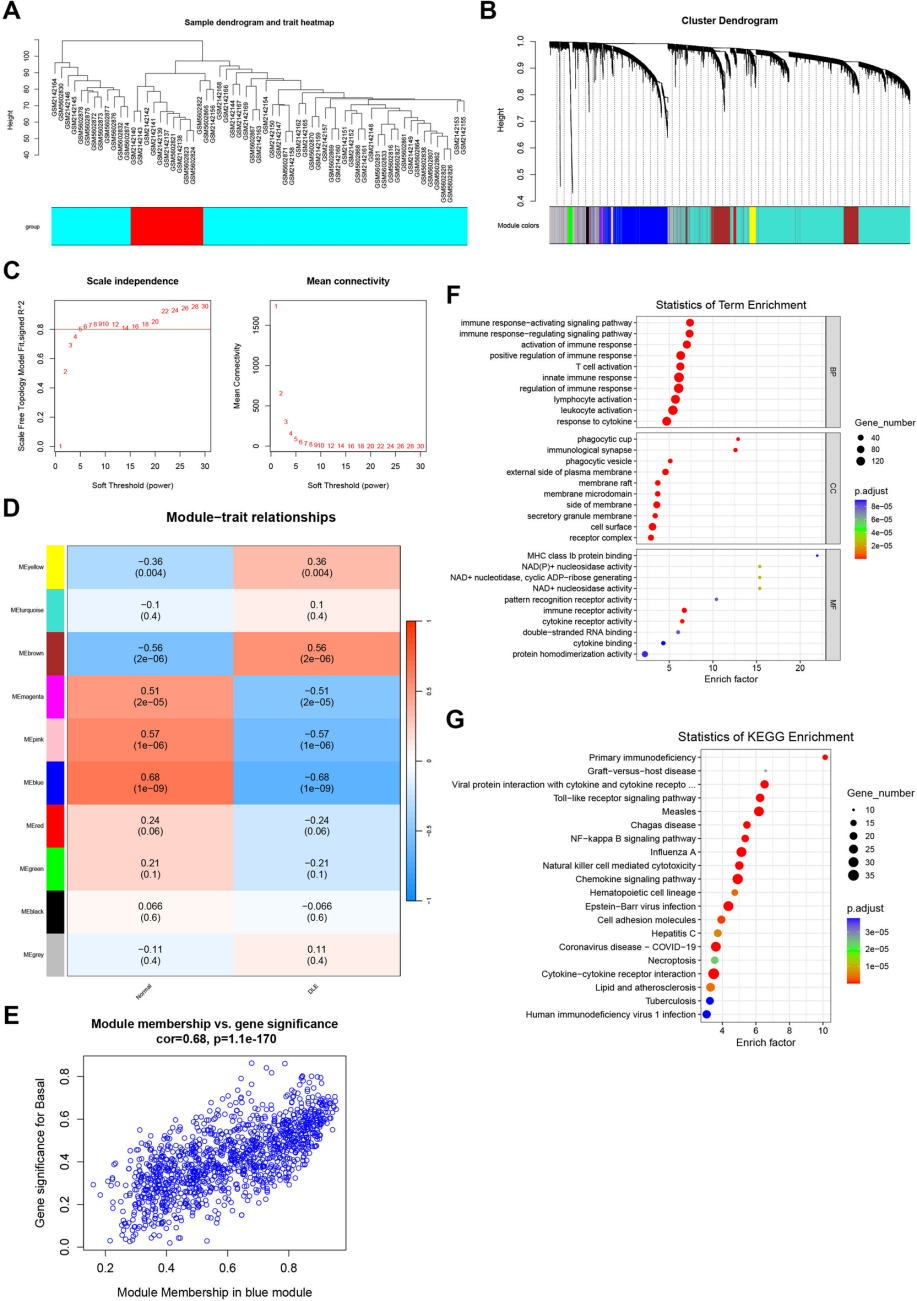
**The WGCNA outcomes based on GSE81071 and GSE184989. A** The dendrogram of all the samples in GSE81071 and GSE184989, presenting the hierarchical clustering outcomes of the samples. **B** The hierarchical clustering dendrogram of co-expressed genes based on the samples in A presents the hierarchical clustering outcomes of the co-expressed genes. **C** The soft-threshold selection graph of WGCNA is employed to select a suitable soft threshold, with the aim of ensuring that the gene network complies with the assumption of scale-free distribution. **D** The module-trait association diagrams between the Normal and DLE groups. Red/positive values signify positive correlations, and conversely, they represent negative correlations. The horizontal axis denotes different phenotypic traits, and the vertical axis indicates modules. Each cell displays the correlation coefficient and P value of the module and the trait. Red/positive values denote positive correlations, and vice versa for negative correlations. **E** The scatter plot of the MM-GS correlation of the MEblue module. **F** A GO enrichment bubble chart was constructed based on the genes of the MEblue module. **G** A KEGG enrichment bubble chart was constructed based on the genes of the MEblue module.

To further narrow the range of key genes of DLE based on the microarray datasets, hereinafter, we merged all samples from the microarray datasets GSE81071 and GSE184989 for WGCNA and categorized the samples into the healthy control skin biopsy group (Normal) and the discoid lupus erythematosus patient skin biopsy group (DLE). The sample clustering tree [Fig. 2A] and the gene clustering tree [Fig. 2B] were employed to present the clustering of the filtered samples and the gene modules. The soft threshold selection plot was utilized to determine β = 5 as the optimal threshold for constructing the scale-free network [Fig. 2C], and the co-expression network was constructed to identify the co-expression gene modules of the Normal and DLE groups. Through the gene module heatmap [Fig. 2D] and the module-trait association plot [Fig. 2E], we discovered that the MEblue gene module exhibited the most significant correlation with both the Normal and DLE groups (cor = 0.68). Subsequently, all the genes encompassed in the MEblue module were extracted [Supplementary Table S3] and underwent GO and KEGG enrichment analyses. The outcomes revealed that the genes in the MEblue module were enriched in GO terms related to immune activation, immune regulation, and T cell activation (such as “immune response - activating signaling pathway”, “immune response - regulating signaling pathway”, “T cell activation”, “lymphocyte activation”) [Fig. 2F] and KEGG terms related to immune deficiency, cytotoxicity, and cytokines (such as “Primary immunodeficiency”, “Chemokine signaling pathway”, “Cytokine - cytokine receptor interaction”) [Fig. 2G].

**Fig. 3:**
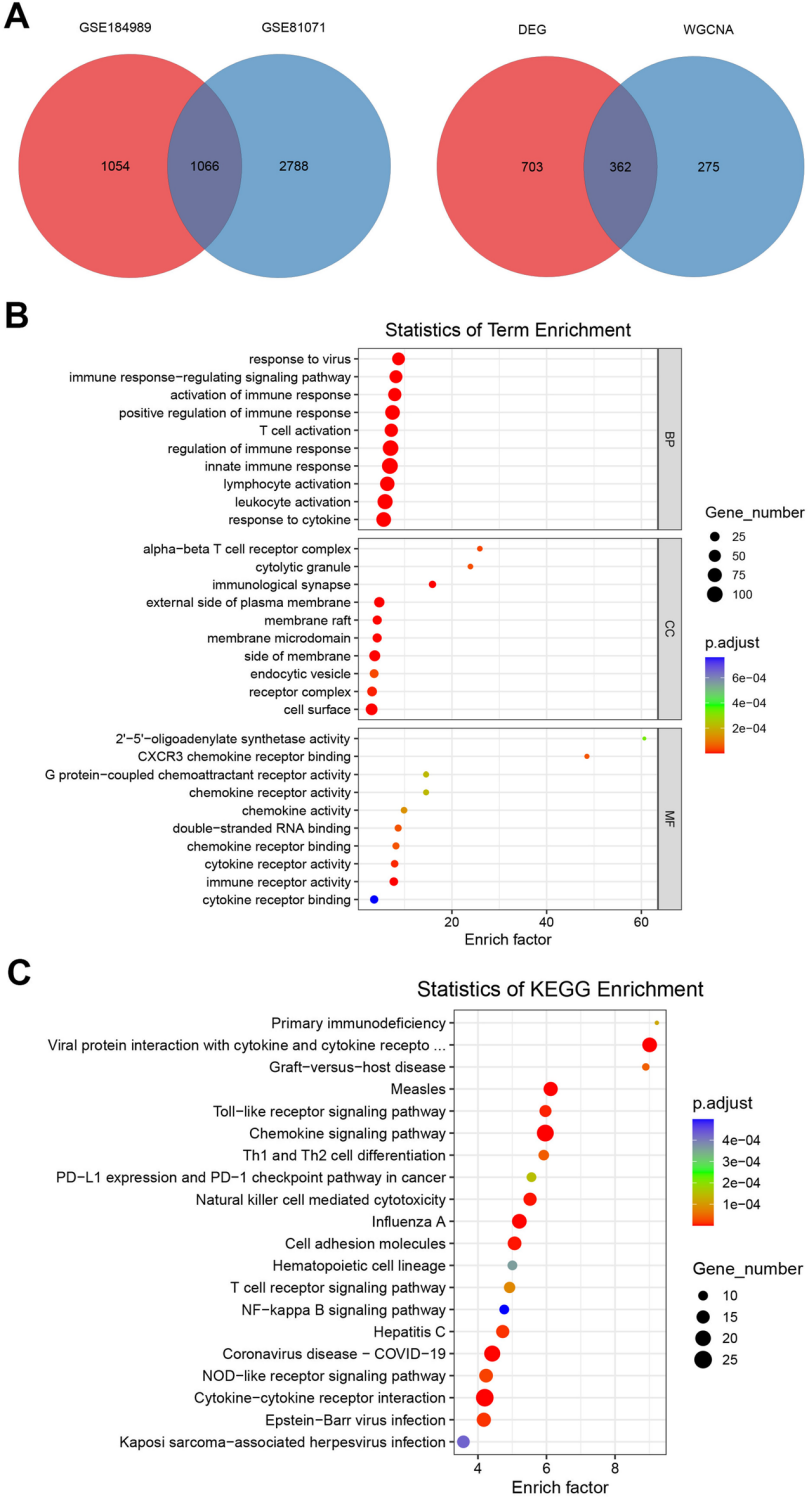
**Intersection analysis of the differentially expressed genes in the transcriptome and the genes in the WGCNA modules. A** The Venn diagram of the differentially expressed genes of SE81071 and GSE184989, among which the intersection genes were again made into a Venn diagram with the genes of the MEblue module in WGCNA. **B** Based on the intersection genes in A, a GO enrichment bubble chart was constructed. **C** Based on the intersection genes in A, a KEGG enrichment bubble chart was constructed.

Subsequently, Venn diagrams were constructed for the differentially expressed genes in the microarray datasets GSE81071 and GSE184989 and the genes contained in the MEblue module of WGCNA [Fig. 3A], obtaining the intersection genes of these three gene sets [Supplementary Tables S4,S5], and GO and KEGG enrichment analyses were performed [Fig. 3B, C]. The enrichment results were centered on T cell-related entries, such as “T cell activation”, “leukocyte activation”, “Th1 and Th2 cell differentiation”, “T cell receptor signaling pathway”. In conclusion, the enrichment analysis of the microarray target gene set after intersection screening indicates that T cells may potentially play a crucial role in the regulation of the immune response in DLE.

### Single-cell sequencing discloses the inflammatory infiltration of T cells in DLE

**Fig. 4:**
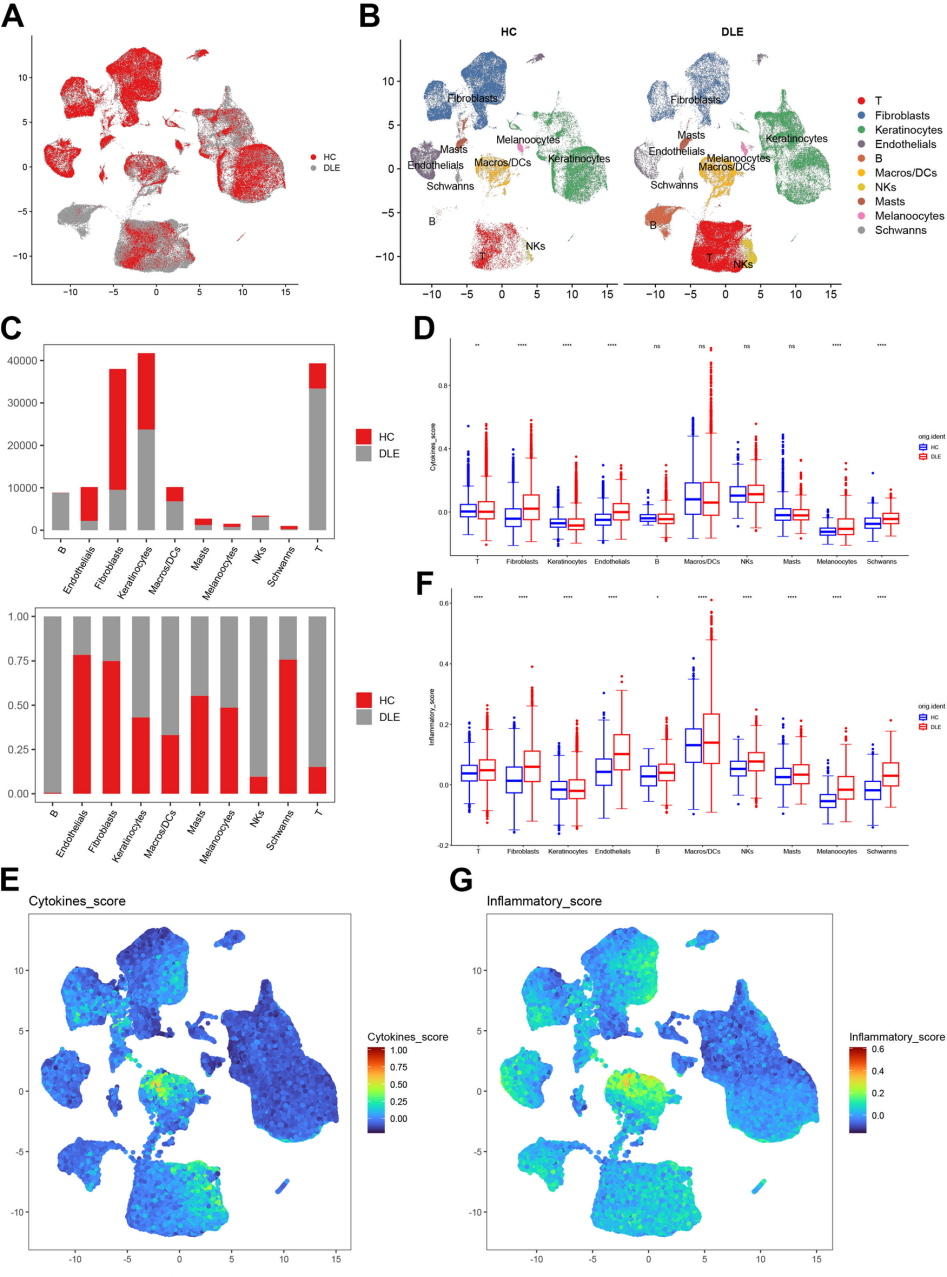
**Analysis of single-cell sequencing data based on GSE179633. A** The initial UMAP clustering graph after sample anchoring. **B** The UMAP clustering graph of cell groups after annotation. **C** Bar chart showing the proportion of cell clusters. **D** Box plots of cytokine scores for each subgroup (**P < 0.01, ****P < 0.0001). **E** Feature plot of cytokine scores for each subpopulation. **F** Box plots of inflammatory factor scores for each subpopulation (*P < 0.05, ****P < 0.0001). **G** Feature plot of inflammatory factor scores for each subpopulation.

To delve profoundly into the significance of T cells in DLE, we propose to isolate T cells from the single-cell sequencing dataset. Based on the single-cell sequencing dataset GSE179633 [Fig. 4A], all cells were categorized into the healthy control skin biopsy group (HC) and the discoid lupus erythematosus patient skin biopsy group (DLE) (the list of differentially expressed genes between the two groups is presented in Supplementary Table S6). We clustered ten cell populations therefrom [Fig. 4B] (the expression map of marker genes for each cell population is depicted in Supplementary Fig. 1). We enumerated the number of cells in these ten cell clusters and calculated the proportion. We discovered that, in alignment with the immune infiltration analysis results of the microarray dataset, the infiltration of T cells in DLE significantly augmented, followed by B cells and macrophages/dendritic cells (Macros/DCs) [Fig. 4C]. Subsequently, we conducted cytokine scoring [Fig. 4D, E] and inflammation scoring [Fig. 4F, G] for each of these ten cell clusters respectively. The outcomes indicated that the cytokine scoring and inflammation scoring of T cells were prominently elevated in DLE [Fig. 4D, F]. Although the cytokine scoring and inflammation scoring of macrophages/dendritic cells manifested high scores in the overall performance of all cell clusters [Fig. 4E, G], the alteration of the macrophages/dendritic cells cluster in cytokine scoring in DLE was not statistically significant [Fig. 4D]. In conclusion, the preliminary analysis of the single-cell sequencing dataset GSE179633 affirmed the conclusions derived from the microarray datasets GSE81071 and GSE184989, jointly uncovering the combined correlation of T cells with cytokines and inflammatory responses in DLE, suggesting that T cells play a crucial role in the immune imbalance of DLE.

### T cells elicit the inflammatory response of DLE through the interaction between external apoptosis and cytokines, and it has been discovered that there exists a T cell in the stress response state (T_STR_)

**Fig. 5:**
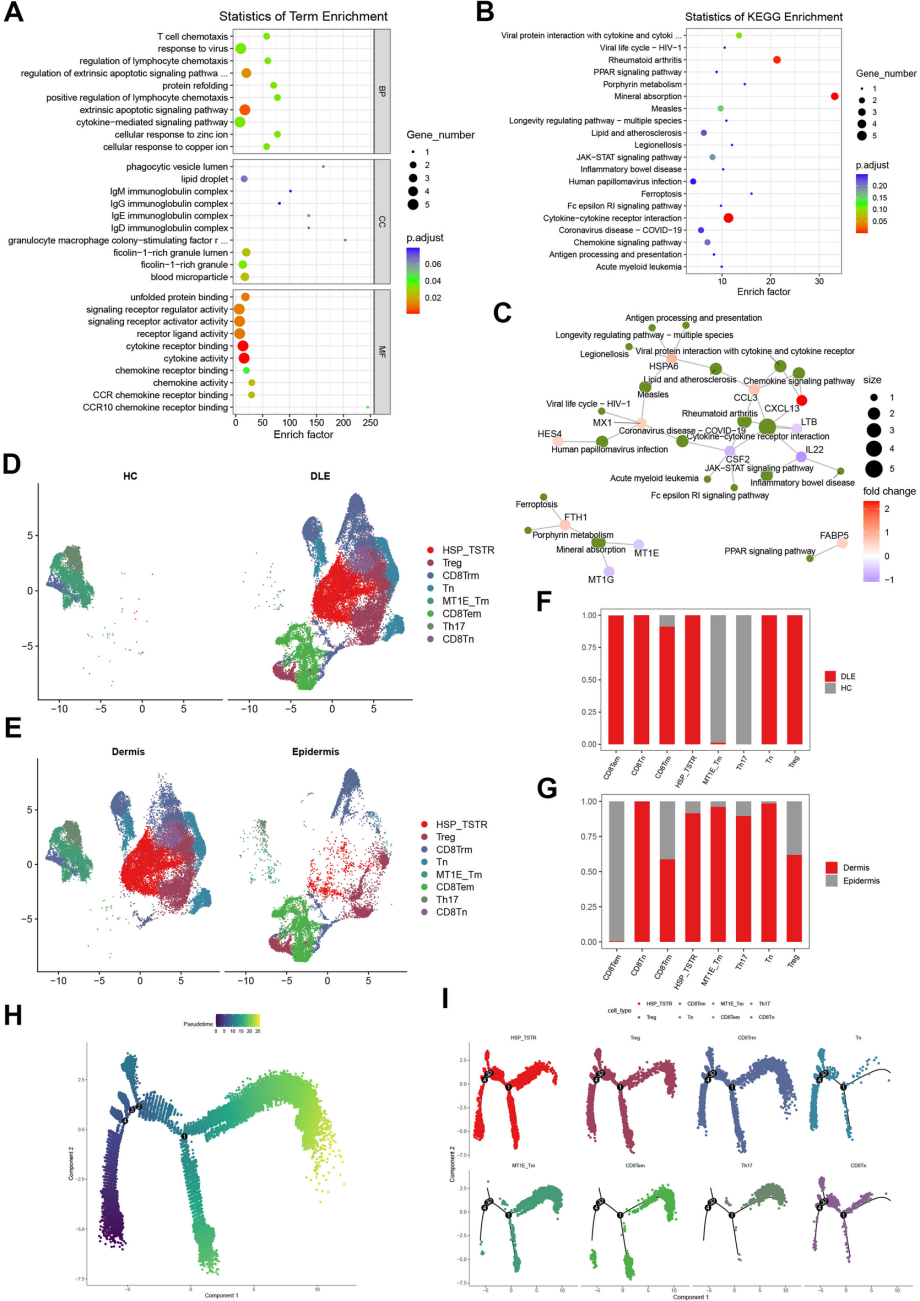
**Re-clustering analysis of T cell clusters within GSE179633. A** Bubble chart of GO enrichment analysis of the differentially expressed genes acquired through the comparison of T cell clusters with other cell clusters. **B** Bubble chart of KEGG enrichment analysis of the differentially expressed genes derived from the comparison of T cell clusters with other cell clusters. **C** The network diagram of pathways and genes in the KEGG enrichment analysis of Group B, wherein green dots signify pathways and dots of other colors represent genes. The color variations of the dots represent the fold change of the genes. **D** T cells were subjected to reclustering and the T cell subsets were annotated (presented in groups of HC and DLE). **E** T-cell re-clustering (presented in groups of Dermis and Epidermis). **F** Bar chart depicting the quantitative proportion of T cell subsets in the HC and DLE groups. **G** Bar chart presenting the quantitative proportion of T cell subsets in the Dermis and Epidermis groups. **H** The distribution of the pseudo-time axis in the pseudo-temporal analysis of T cell clusters. **I** With each T cell cluster as a unit, separate and present the pseudo-temporal distribution of cells in each subpopulation.

Subsequently, to comprehensively elucidate the alterations in the T cell landscape within DLE, we carried out an analysis centered on the T cell clusters in the single-cell sequencing dataset GSE179633. First, a cluster-based differential analysis was performed on these 10 cell clusters to extract the differentially expressed genes of the T cell clusters [Supplementary Table S7], and GO and KEGG enrichment analyses were conducted on these differentially expressed genes [Fig. 5A-C]. The results of GO enrichment revealed that the molecular functions of the differentially expressed genes in the T cell clusters were most prominently represented in cytokine activity and cytokine receptor binding. Simultaneously, the most salient biological processes were concentrated in the extrinsic apoptotic signaling pathway. In conjunction with the most significant Cytokine-cytokine receptor interaction pathway in the KEGG enrichment results, all these enrichment outcomes indicated that T cells initiate inflammatory responses through external apoptosis and the interactions between cytokines. Furthermore, the KEGG network diagram also demonstrated that CXCL13 was the gene with the most significant fold change among those related to the Cytokine-cytokine receptor interaction pathway and the Chemokine signaling pathway.

Thereafter, we isolated the T cell clusters and performed re-clustering. Cell annotation was conducted in accordance with the marker genes reported in certain existing literature [Supplementary Fig. 2]. Eventually, eight T cell clusters were annotated. The UMAP plots depicting the distribution of cell populations were presented in two categorization frameworks: one based on HC and DLE, and the other on the dermis and epidermis layers [Fig. 5D, E], namely, stress response state T cells (HSP_TSTR), regulatory T cells (Treg), CD8(+) tissue-resident memory T cells (CD8Trm), naive CD4(+) T cells (Tn), naive CD8(+) T cells (CD8Tn), MT1E(+) memory T cells (MT1E_Tm), CD8(+) effector T cells (CD8Tem), and helper T cells 17 (Th17). In combination with the bar chart of the proportion of T cells [Fig. 5F, G], it can be observed that a considerable number of T cells infiltrate in DLE. With the exception of the MT1E_Tm and Th17 clusters, the extensive infiltration of the remaining T cell clusters mainly occurs in the DLE [Fig. 5F]. These T cell populations, which are predominantly present in DLE (CD8Tem, CD8Tn, CD8Trm, HSP_TSTR, Tn, Treg), exhibit distinct infiltration characteristics in the dermis and epidermis. Specifically, CD8Tem primarily infiltrates the epidermis, CD8Tn and Tn mainly infiltrate the dermis, and CD8Trm, HSP_TSTR, and Treg infiltrate both the dermis and the epidermis [Fig. 5G]. Next, we carried out pseudo-temporal analysis on the T cell clusters [Fig. 5H]. From the pseudo-temporal process of each T cell cluster, CD8Tem predominantly accumulates at the terminal stage of the pseudo - temporal trajectory, whereas CD8Tn and Tn mainly aggregate in the early and middle segments of the pseudo - temporal trajectory. This manifestation aligns with the invasive characteristics of DLE from the dermis to the epidermis, indicating that the results of the pseudo - temporal analysis possess reliable reference significance. In contrast to CD8Tem, CD8Tn, and Tn, HSP_TSTR, Treg, and CD8Trm are distributed across the entire pseudo - temporal interval. This suggests that HSP_TSTR, Treg, and CD8Trm play a more comprehensive and potential role in DLE [Fig. 5I]. In conclusion, via a comprehensive dissection of the T - cell atlas, we herein report a stress - responsive state T cell (T_STR_) infiltrating within DLE, and delve into its distribution characteristics and potential importance.

### The expression of key genes associated with T cells is increased in DLE, and the high expression of CXCL13 and GNLY is a unique feature of DLE

**Fig. 6:**
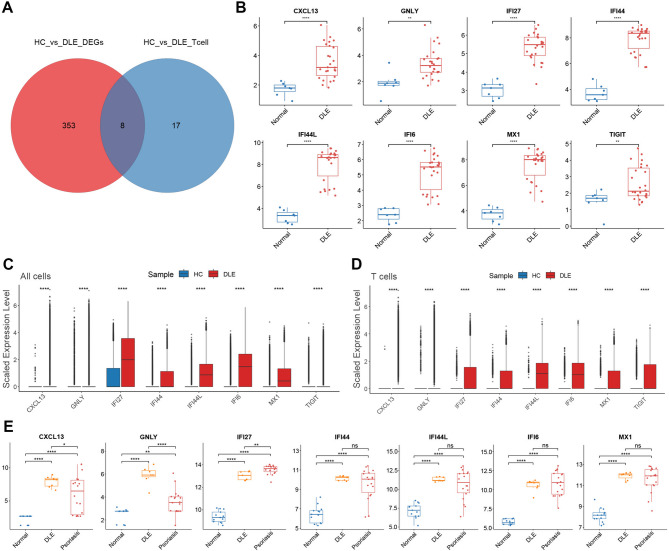
**The key genes related to T cells in DLE. A** Integrate the DLE differentially expressed genes [Supplementary Table S6] and T cell differentially expressed genes [Supplementary Table S7] from GSE179633 to create a Venn diagram. **B** Box plots of the expressions of CXCL13, GNLY, IFI6, IFI27, IFI44, IFI44L, MX1, and TIGIT in GSE81071. **C** Box plots of gene expression of CXCL13, GNLY, IFI6, IFI27, IFI44, IFI44L, MX1, and TIGIT based on all cells in GSE179633. **D** Box plots of gene expression of CXCL13, GNLY, IFI6, IFI27, IFI44, IFI44L, MX1, and TIGIT based on T cells in GSE179633. **E** Box plots of gene expression of CXCL13, GNLY, IFI6, IFI27, IFI44, IFI44L, MX1, and TIGIT in GSE52471.

To further narrow the scope of genes that play a crucial role in T cells, we constructed a Venn diagram [Fig. 6A] of the differentially expressed genes between the HC and DLE groups [Supplementary Table S6] and the T cell-specific genes [Supplementary Table S7] in the single-cell sequencing dataset GSE179633, and extracted the 8 overlapping genes, namely CXCL13, GNLY, IFI6, IFI27, IFI44, IFI44L, MX1, and TIGIT. We verified the expression levels of these genes in GSE81071 [Fig. 6B], GSE184989 [Supplementary Fig. 4 A], and GSE179633 [Fig. 6C, D] [Supplementary Fig. 4B], respectively. The results consistently demonstrated that these key genes were highly expressed at the overall cellular level in DLE.

To determine whether these key genes are exclusive to DLE, we validated the expression of these crucial genes within the transcriptome dataset GSE52471, which includes psoriasis samples [Fig. 6E]. The findings indicated that there were significant differences in the expression levels of CXCL13, GNLY, and IFI27 between the DLE and psoriasis. Specifically, the high expression of CXCL13 and GNLY is a characteristic unique of DLE. Among them, CXCL13 was previously discovered to be associated with the Cytokine-cytokine receptor interaction pathway and the Chemokine signaling pathway in the KEGG analysis based on T cell-specific genes.

## Discussion

In our research, through the combined analysis of transcriptome data, T cells were ultimately determined as the major infiltrating immune cell cluster in the lesion site of DLE. Subsequently, through more in-depth studies such as single-cell sequencing analysis and re-clustering based on T cells, a type of stress response state T cells was discovered among the infiltrating T cells in DLE. Eventually, by performing overlapping statistics on the key gene lists obtained from numerous key analysis steps, CXCL13, GNLY, IFI6, IFI27, IFI44, IFI44L, MX1, and TIGIT were extracted as the key genes associated with the infiltrating T cells.

C-X-C Motif Chemokine Ligand 13 (CXCL13) can be generated by mesenchymal lymphoid tissue histiocytes, follicular dendritic cells, and human follicular helper T cells^23^. Research has unveiled the significant role of the binding of CXCL13 to its receptor CXCR5 in autoimmune diseases (concerning inflammation regulation and immune response)^24^. For instance, in systemic lupus erythematosus (SLE), it has been found that CXCL13 promotes the proliferation of mesangial cells through binding to CXCR5^25^. Meanwhile, another study has disclosed that the CD4 T cell phenotype in SLE patients is imbalanced, manifested as the expansion of PD-1+/ICOS + CXCL13 + T cells^26^. Furthermore, the CXCL13-CXCR5 signaling axis is regarded as a key mechanism in multiple inflammatory disorders, such as neuroinflammation^27,28^, interstitial cystitis/bladder pain syndrome^29^, and renal transplant inflammation^30^, among others. In our study, CXCL13 exhibited a consistently high expression in the DLE transcriptome data as in these inflammatory diseases, indicating the pivotal role of CXCL13 as an inflammatory chemokine in the onset and progression of DLE inflammation. This discovery also relates to a finding from a study suggesting that the systemic upregulation of CXCL13 is mainly driven by peripheral helper T (Tph) and regulatory T (Treg) cells^31^, which is in accordance with the extensive infiltration of Treg cells in DLE as analyzed by us. Additionally, in our research outcomes, CXCL13 possesses a high T cell specificity, suggesting that CXCL13 is inseparably associated with T cells in the pathogenesis of DLE, which will be a potential subject for our future follow-up studies. Furthermore, CXCL13, an inflammatory chemokine that regulates the migration of B cells, can mediate the functional interaction between B cells and T cells. Through the analysis of single-cell transcriptome datasets, this study revealed a high degree of B-cell infiltration in DLE lesion tissues. This finding further validates the crucial involvement of B cells in the inflammatory pathological mechanism of DLE. Existing research has also indicated that DLE exhibits more prominent B-cell transcriptional profiles in skin tissues and demonstrates more significant B-cell enrichment in immunohistochemical staining experiments^13^. In this study, following the differential enrichment analysis of the B-cell population [Supplementary Fig. 3B], we observed that the biological functions and signaling pathways associated with B cells are closely functionally related to those of T cells. In summary, there is clear experimental evidence for the biological significance of CXCL13 as a characteristic key gene in DLE. It can act as a functional molecular bridge, connecting the two core effector cells, B cells and T cells, during the humoral immune response.

The protein encoded by the Granulysin (GNLY) gene is a member of the saposin-like protein (SAPLIP) superfamily. It is a well-established marker of cytotoxic molecules and is primarily localized within the cytotoxic granules of T cells. When T cells are stimulated by antigenic signals, these granules are released in a targeted fashion. Multiple studies have confirmed that cytotoxic granules, with GNLY being a typical example, play a crucial role in the onset and progression of lupus diseases^32,33^. Notably, aside from T cells, research reports have indicated that in patients with systemic lupus erythematosus (SLE), the transcriptional expression levels of cytotoxic effector factors centered around GNLY in B cells are also significantly upregulated^34^. This study had the core objective of screening for T-cell-associated specific genes and concurrently incorporated a psoriasis transcriptome dataset for cross-validation. The findings demonstrated that GNLY not only exhibits clear T-cell expression specificity but also represents a key gene with specifically high expression in DLE. Integrating the above-mentioned published research findings, it can be further deduced that cytotoxic effector factors typified by GNLY may play a central mediating role in the regulation of the inflammatory pathological mechanism of DLE. Additionally, this molecule holds promise as a functional molecular bridge that links the coordinated functions of B cells and T cells.

Interferon-induced protein 44 (IFI44) and interferon-induced protein 44-like (IFI44L) are interferon-stimulated genes (ISGs), whose status as key biomarkers in SLE has been confirmed through research^35^. Moreover, IFI44L has been formally recognized as a key hub gene in SLE^6,36^, and it has been disclosed that the methylation level of IFI44L can be utilized to DLE from SLE^15^. IFI44 and IFI44L have also been identified as key hub genes in several inflammatory diseases, such as influenza^37^, dermatomyositis^38^, and COVID-19 infection^39^, among others. In existing bioinformatics analyses related to DLE, IFI44 has also been enlisted as a key regulatory gene for DLE^40^. In the screening outcomes of key genes related to infiltrating T cells in DLE in our study, IFI6 and IFI27, being interferon-stimulated genes as well, have been regarded as closely associated with the pathogenesis of SLE/DLE in multiple SLE/DLE bioinformatics studies^41–43^. Myxoma resistance 1 (MX1) is a type I interferon-stimulated gene. Numerous studies have demonstrated that MX1 possesses significant anti-infection and antiviral functions^43,44^, and the gene programming regulation of the antiviral state of T cells is intimately related to MX1^45^. MX1 has been considered one of the hub genes in the pathogenesis of DLE^41^, and there are also studies on SLE that have identified MX1 as a potential biomarker for diagnosing SLE in peripheral blood^46^.

The definition of the stress response state T cell (T_STR_) stems from a pan-cancer T cell atlas study^47^, in which it was elucidated that T_STR_ is a group of T cells that have been in a persistent stress state, primarily expressing stress-related genes such as heat shock protein genes. In this study, the dysfunction of this type of T cell was mainly manifested as the loss of sensitivity to anti-tumor drugs and the development of drug resistance. Nevertheless, as of now, the majority of research on T_STR_ pertains to cancer, and studies in autoimmune diseases remain to be ventured into. Despite the identification of the existence of T_STR_ in our study, the role played by T_STR_ in DLE, its interaction with other cells, the implicated mechanisms, and whether new therapeutic approaches can be developed therefrom still constitute directions that require further exploration subsequent to this study.

This study further corroborates that fibroblasts, keratinocytes, and endothelial cells are all implicated in the inflammatory pathological progression of DLE. Their roles can be validated by the significantly elevated inflammatory response scores and cytokine expression levels. Indeed, the functions of these cells in the pathological mechanism of DLE have already received partial attention in previous research. Among them, fibroblasts have been demonstrated to participate in the development of various skin diseases and autoimmune disorders through mechanisms such as secreting pro-inflammatory factors and mediating antigen presentation^48^. The pathogenic effects of keratinocytes in lupus diseases have also become increasingly clear. These cells are not only capable of synthesizing and releasing type I interferon (IFN-I), thereby triggering a series of downstream immune-inflammatory cascade reactions^49^, but they can also form a regulatory circuit with fibroblasts. By recruiting myeloid cells, they further amplify the IFN-I-driven autoimmune inflammatory response^50^. Additionally, once activated within the inflammatory microenvironment, endothelial cells can promote the migration and infiltration of immune cells to the sites of skin inflammation by upregulating the expression of adhesion molecules and enhancing vascular permeability. In conclusion, the intricate interplay and regulatory mechanisms between these non-immune cells and immune cells collectively constitute the complex pathological mechanism network of DLE. Nevertheless, the specific regulatory pathways and molecular mechanisms underlying this process still necessitate further in-depth investigations for a comprehensive elucidation.

In conclusion, through the combined analysis of transcriptome data and single-cell sequencing data, we demonstrated the high infiltration of T cells in DLE, as well as the significantly elevated inflammation-related and cytokine-related features. Furthermore, we uncovered an innovative discovery that a considerable number of stress response state T cells are present in DLE. This finding may direct new research directions for the pathogenesis of DLE in the future or offer assistance in the development of DLE biomarkers and therapeutic targets. Furthermore, in the systematic analysis of T-cell-associated differential genes in this study, following multiple rounds of rigorous screening and validation, CXCL13, GNLY, IFI6, IFI27, IFI44, IFI44L, MX1, and TIGIT were eventually identified as crucial disease genes in DLE that are closely associated with the T -cell regulatory mechanism. To further validate the disease-specific nature of the aforementioned genes, the research team incorporated a psoriasis dataset for cross-validation. The results indicated that CXCL13 and GNLY displayed a characteristic high-expression pattern in DLE samples that was significantly higher than that in psoriasis samples. This finding further clarifies the potential specific roles of these two genes in the pathological progression of DLE.

### Limitations of the study

The omics data utilized in this research were all obtained from the open-source data of the Gene Expression Omnibus database. Nevertheless, it is important to note that the samples in the two transcriptome datasets and one single-cell high-throughput sequencing dataset employed in the study did not originate from the same individual. The variations among the samples resulted in inconsistent expression trends of some genes in the transcriptome data and the single-cell high-throughput sequencing data.

## Supplementary Information

Below is the link to the electronic supplementary material.

Supplementary Material 1

Supplementary Material 2

Supplementary Material 3

Supplementary Material 4

Supplementary Material 5

Supplementary Material 6

## Data Availability

The transcriptome data were deposited in the Gene Expression Omnibus database under accession numbers GSE81071, GSE184989 and GSE52471, and are accessible at the following URLs: https://www.ncbi.nlm.nih.gov/geo/query/acc.cgi? acc=GSE81071, https://www.ncbi.nlm.nih.gov/geo/query/acc.cgi? acc=GSE184989 and https://www.ncbi.nlm.nih.gov/geo/query/acc.cgi? acc=GSE52471. The single-cell RNA sequencing data were deposited in the Gene Expression Omnibus database under accession number GSE179633, and are accessible at the following URL: https://www.ncbi.nlm.nih.gov/geo/query/acc.cgi? acc=GSE179633.

## Declarations

### Supplemental information

This study includes 4 supplementary figures and 8 supplementary tables.
