# Supplementary material for: Identification of T cell stress response state (TSTR) and key genes related to T cells in discoid lupus erythematosus
Source: Sci Rep. 2025 Nov 10;15:39262. doi: 10.1038/s41598-025-23088-7 (PMC12603320; doi:10.1038/s41598-025-23088-7)
Supplement: Supplementary file 6 — Supplementary Material 6 [file 41598_2025_23088_MOESM6_ESM.docx]

**Legends for supplementary figures and tables**

Supplementary Fig.1 is a complementary file to Fig. 4, which shows the feature plots of marker genes for each cell population.

Supplementary Fig.2 is a supplementary document for Fig. 5, depicting the dot plot of marker gene expressions in the initial unannotated re-clustering of T cells.

Supplementary Fig.3 illustrates the results of GO and KEGG enrichment analyses for macrophages/dendritic cells (Macros/DCs) (Panel A) and B cells (Panel B) within the GSE179633 dataset.

Supplementary Fig.4 is a complementary file for Fig. 6, demonstrating the box plots of the expressions of CXCL13, GNLY, IFI6, IFI27, IFI44, IFI44L, MX1, and TIGIT in GSE184989 as well as the feature plots of their expressions in GSE179633.

The Supplementary Table integrates Supplementary Table S1 to Supplementary Table S8. Supplementary Table S1 to Supplementary Table S7 exhibit the gene lists of each analytical link. Supplementary Table S8 compiles the analytical software employed in this research, together with their version numbers and download link addresses.
